# Supplementary material for: Cross-neutralization of SARS-CoV-2 by HIV-1 specific broadly neutralizing antibodies and polyclonal plasma
Source: PLoS Pathog. 2021 Sep 24;17(9):e1009958. doi: 10.1371/journal.ppat.1009958 (PMC8494312; doi:10.1371/journal.ppat.1009958)
Supplement: Attachment — Submitted filename: 115362_1_rebuttal_2141138_qx6c5c.docx [file ppat.1009958.s001.docx]

**Response to Reviewers**

We thank the reviewers for their valuable and pertinent suggestions that helped us immensely in revising our manuscript. Listed below are the responses to reviewers’ comments where reviewer’s comments are highlighted in bold while the responses are in normal font.

**Reviewer #1 (Evidence, reproducibility and clarity (Required))**

**As HIV-1 bnAbs show extensive somatic mutations and have several unique properties such as long CDR and ability to recognize glycopeptides, there is a possibility that some of them can cross-react with SARS-CoV-2 and a fraction of these could even neutralize the virus. To explore if this is true, Mishra et al from the Luthra Lab study investigated the cross-reactivity of existing HIV-1 neutralizing antibodies with purified and stabilized SARS-CoV-2 trimeric ectodomain S2P and receptor binding domain. Indeed, they find that antibodies that recognize the CD4 binding site and MPER region of HIV-envelope show fairly strong reactivity to SARS-CoV-2 RBD and S2P like the CoV-1 cross reactive antibody CR3022. They further go on to test neutralization using pseudoviruses decorated with SARS-CoV-2 spike and find that one of the several binding antibodies, N6 can indeed neutralize, though not potently as expected. They also assessed the cross-reactivity and neutralization of plasma from children with chronic HIV-1 infection to SARS-CoV-2 pseudoviruses. Some sample of plasma indeed showed neutralization of SARS-CoV-2 pseudoviruses. Based on these observations, the authors propose cross-reactive epitopes such as the N6 epitope can be used as a blueprint to engineer variant super-antibodies that can be effective against several viral pathogens including SARS-VoV-2. Overall, the study is well conducted and detailed with clarity.**

**Q1. The authors should include an authentic SARS-CoV-2 neutralizing antibodies in the study as a positive control. There are several RBD as well as NTD binding nAbs that have been identified. This will allow one to assess the extent of cross-reactivity and neutralization exhibited by HIV-1 bnAbs and gauge the extent of practical utility. CR3022 while important, is not enough.**

R1. We have now included an Anti-RBD nAb, CC12.1, as positive control for both binding assay and neutralization assays (**line numbers 73 – 77**).

**Q2. Some plasma of HIV-1 infected children showed neutralizing activity against SARS-CoV-2. However, the neutralization seen may not be solely due to HIV-1 bnAbs in plasma. Antibodies to other human coronaviruses likely contributes to this neutralization. The authors should also test reactivity/neutralization to 229E, NL63, OC43, HKU1, MERS spike/pseudoviruses to support their claim. In its absence, the authors should tone down their interpretation and provide alternate explanation.**

R2. We have now performed neutralization assays with the plasma from all the HIV-1 infected children against all seven coronaviruses (SARS-CoV-1, SARS-CoV-2, HKU1, NL63, MERS, OC43, and 229E) (**line numbers 150 – 160 and 551 – 564**).

**Q3. The authors start out with the following premise "Given the unique nature of HIV-1 bnAbs and their ability to recognize and/or accommodate viral glycans, we reasoned that the glycan shield of SARS-CoV-2 spike protein can be targeted by HIV-1 specific bnAbs". While HIV-1 envelope is heavily glycosylated, it is interesting to note that the cross-reactive HIV-1 bnAbs target RBD of SARS-CoV-2 spike protein which is not heavily glycosylated (2 sites). In absence of glycosylations-probing experiments, it is difficult to justify this premise. Rather the extensive SHM and self-reactivity (MPER-directed antibodies) could be the likely reason for cross-reactivity.**

R3. We have discussed the polyreactivity and/or autoreactivity of HIV-1 bnAbs as one of the plausible reasons for the observed cross-reactivity with SARS-CoV-2 (**line numbers 166 – 180**).

**Minor**

**Q4. The color profile of in figure 3 needs to be revised or symbols added to allow the curves to be distinguished from each other.**

R. We have changed the color profile of figure 3 and have added symbols as well (**line number 527**). Figure 3 from the original manuscript is now figure 4 in the revised manuscript.

**Reviewer #2 (Significance (Required))**

**This is a significant study that highlights the concept of developing a broadly cross-reactive antibodies that may be effective against multiple pathogens, a concept not usually ascribed to antibodies that are known for their specificity unlike drugs that can be repurposed. In-depth study of epitopes that can elicit multi-pathogen cross-reactive/neutralizing antibodies can on the other hand allow generation of antigenic templates that can be effective as vaccines. This study is suitable for broader audiences to disseminate above-mentioned concepts.**

****Referee Cross-commenting****

**Q1. There is a lot of evidence now that lenti-pseudoviruses are a good surrogate for identifying SARS-CoV-2 neutralizing antibodies. That said the authors may want to ensure the data is reproducible even with authentic SARS-CoV-2 viruses as reviewer 2 suggests to avoid surprises.**

R1. We have now tested the ability of N6 to neutralize authentic SARS-CoV-2 virus using a cytopathic effect (CPE) based neutralization assay. N6 failed to show any reduction in CPE upto a concentration of 20 µg/ml (**line numbers 135 – 141**).

We have now discussed N6’s inability to neutralize authentic virus in discussion section (**line numbers 181 – 187**).

**Q2. I also agree with the Reviewer 2 that structural analyses of the cross-neutralizing antibody with SARS-CoV-2 spike will strengthen the manuscript.**

R. While we do agree with both the reviewer’s suggestion that a structural analysis of N6 binding with SARS-CoV-2 will provide details into the exact nature of epitopes-paratopes that are responsible for the cross-reactivity observed, we believe it to be out of scope for the current work. The current work was designed to show the ability of polyreactive and somatically hypermutated antibodies generated in chronic HIV-1 infection to bind to new and emerging pathogens such as SARS-CoV-2. While only N6 showed neutralization of SARS-CoV-2, it is also noteworthy that several HIV-1 bnAbs showed significant binding to both purified RBD as well as surface expressed spike from SARS-CoV-2. As all these distinct bnAbs have different paratopes, a detailed structural analysis to understand the exact nature by which several distinct bnAbs cross-reacted with SARS-CoV-2 is not feasible. We are optimistic that our results will provide that impetus for future detailed structural studies (**line numbers 194 – 198**).

**Q3. Also, the authors may want to avoid 293T-ACE2 cells as their targets for carrying out neutralization assays. There is data now from Davide Corti's group that indicate ACE2 overexpression can significantly underestimate the neutralizing capacity of antibodies especially those that bind to RBDs (bioRxiv 2021.04.03.438258; doi: https://doi.org/10.1101/2021.04.03.438258). This may be particularly true for N6. A cell line like Vero E6 that endogenously express ACE2 would be the way to go. If the authors decide to use Lenti pseudoviruses as the first step in Vero cells, please make sure to use G89V mutated HIV gag-pol to avoid TRIM5alpha restriction.**

R. Based on reviewer’s suggestion, we have now performed neutralization assays for all bnAbs using both HEK293T/ACE2 cells and Vero-E6 cells. We have used an HIV-1 proviral backbone that does contain G89V mutation in the gag-pol region (**line numbers 96 – 121**).

**Reviewer #3 (Evidence, reproducibility and clarity (Required))**

**The authors report that HIV-1 sp-bnMAb cross-neutralized SARS-CoV-2 pseudotyped virus in vitro. The finding that N6 can block HIV-based-SARS-CoV-2 pVs from entering 293T-ACE2 cells is intriguing and requires further investigation before the manuscript can be considered for publication.**

**Major comments**

**Q1. The authors must demonstrate that N6 is capable of neutralizing authentic SARS-CoV-2.**

R1. We have now tested the ability of N6 to neutralize authentic SARS-CoV-2 virus using a cytopathic effect (CPE) based neutralization assay. N6 failed to show any reduction in CPE upto a concentration of 20 µg/ml (**line numbers 135 – 141**).

We have now discussed N6’s inability to neutralize authentic virus in discussion section (**line numbers 181 – 187**).

**Q2. The authors should also demonstrate it works in another system i.e., recombinant VSV/SARS-CoV-2 (used by Naveenchandra Suryadevara et al., 2021 "Neutralizing and protective human monoclonal antibodies 1 recognizing the N2 terminal domain of the SARS-CoV-2 spike protein" https://doi.org/10.1016/j.cell.2021.03.029) which is a well validated system for SARS-CoV-2 neutralization activity.**

R2. We have additionally used a MLV based pseudovirus system to further increase the robustness of neutralization assays. In addition, we have now used three distinct cell lines (HEK293T/ACE2, Vero-E6 and Huh7 cells) to exclude any bias in neutralization assays due to endogenous overexpression of ACE2 on HEK293T cells (**line numbers 96 – 121**).

**Q3. Please, reference the HIV-1 based SARS pVs neutralization assay used in this work if it has been used earlier, either by your team or others. Details regarding the performance of this particular assay for SARS-CoV-2 Nab detection will aid the reader to understand its robustness and weaknesses.**

R3. We have provided several references for both the HIV-1 lentiviral and MLV retroviral pseudovirus neutralization assays. Furthermore, we have added detailed results for optimization of neutralization assays that we performed (**line numbers 96 – 121 and figure 3**). In addition, we have discussed the limitation and weaknesses of pseudovirus neutralization assays in comparison to authentic virus neutralization assays (**line numbers 183 – 187**).

**Q4. In the method section, a list of all controls used to guarantee neutralization assay performance must be included (i.e., positive and negative mAb controls, virus controls, etc.); additionally, it might be necessary to show raw data of all control results.**

R4. We have provided the detail of all controls that were used to guarantee the robustness of neutralization assay in the methodology section of “neutralization assay.’ Furthermore, we have now added an entire section in the results titled, ‘**Optimized conditions for neutralization of pseudotyped coronaviruses,’** where we now provide all the technical details that were optimized to ensure the robustness of the neutralization assay (**line numbers 96 – 121**).

**Q5. Control description are missing in the method section for all or most of the assays (some descriptions are found in the result section, but definitely they should be found in M&M, for each technique).**

R5. We have now provided the description for all the controls in methodology section too.

**Q6. The authors draw some similarities between HIV-1 Env and SARS-CoV-2 Spike proteins from structure and glycosylation point of view, but the fact that N6 binds to ENV-CD4bs is beyond these pictures and for that reason a detailed structural analysis MUST be performed to show the mechanism of cross-neutralization and corroborate it exists.**

R6. While we do agree with both the reviewer’s suggestion that a structural analysis of N6 binding with SARS-CoV-2 will provide details into the exact nature of epitopes-paratopes that are responsible for the cross-reactivity observed, we believe it to be out of scope for the current work. The current work was designed to show the ability of polyreactive and somatically hypermutated antibodies generated in chronic HIV-1 infection to bind to new and emerging pathogens such as SARS-CoV-2. While only N6 showed neutralization of SARS-CoV-2, it is also noteworthy that several HIV-1 bnAbs showed significant binding to both purified RBD as well as surface expressed spike from SARS-CoV-2. As all these distinct bnAbs have different paratopes, a detailed structural analysis to understand the exact nature by which several distinct bnAbs cross-reacted with SARS-CoV-2 is not feasible. We wish that our results will provide that impetus for future detailed structural studies (**line numbers 194 – 198**).

**Q7. As the authors may realize, based on previous comments, any false positive results that could have biased the main conclusion of this article, must be excluded in order to claim such intriguing finding.**

Wherever applicable, we have tried to use proper positive and negative control and have tried to tone down the inferences.

**Q8. Regarding BINDING AND NEUTRALIZATION OF SARS-COV-2 WITH PLASMA FROM HIV-1 INFECTED PAEDIATRIC PATIENTS, how can you discard previous exposure to actual SARS-CoV-2 in such population, and/or previous infection with other common hu-CoVs, which has been shown to elicit cross reactive SARS-CoV-2 Abs.**

R. All the pediatric plasma samples used in the binding and neutralization assays were pre-pandemic (2013-2014). While we do agree that exposure to common endemic coronaviruses might have elicited some degree of cross-reactive SARS-CoV-2 antibodies, we have now performed neutralization assay for all seven coronaviruses (including common endemic coronaviruses HKU1, OC43 and 229E) using the plasma that showed SARS-CoV-2 neutralization (**line numbers 150 – 160 and figure 6**).

**Minor comments can be addressed once these main concerns are solved.**

**Reviewer #3 (Significance (Required)):**

**Q9. The fact that N6 (and plasma from HIV-1 infected patients) can neutralize SARS-CoV-2 is novel and intriguing; however, these observations must be supported with new experiments. Showing that N6 neutralizes authentic SARS-CoV-2 would be a key point to address. Moreover, the mechanism for such interaction should be explored using a detailed structural analysis.**

R10. Please see response to query 6.

****Referee Cross-commenting****

**I am happy to realize that there is an agreement in the major points observed by both Reviewers. These comments must be answered properly by the authors now.**
